# Supplementary material for: The association of red and processed meat with gestational diabetes mellitus: Results from 2 Canadian birth cohort studies
Source: PLoS One. 2024 May 30;19(5):e0302208. doi: 10.1371/journal.pone.0302208 (PMC11139301; doi:10.1371/journal.pone.0302208)
Supplement: S1 Fig — (DOCX) [file pone.0302208.s001.docx]

S1 Fig. Sample Population CONSORT Flowchart

**START**

N = 1,012

N

N= 857

**Population for Analysis**

n = 976

**Excluded = 36**

Mothers of multiples, n = 0

Early withdrawal, n = 2

History of Diabetes, n = 4

Age >40, n = 4; Age<18, n = 0

Caloric Intake missing or outside [500,4500], n = 27

**FAMILY**

N = 857

N

N= 857

**Population for Analysis**

n = 581

**Excluded = 276**

Mothers of multiples, n = 41

Non-white ethnicity, n = 133

History of Diabetes, n = 37

Age >40, n =29; Age<18, n =5

Caloric Intake missing or outside [500,4500], n = 92

Note:

OGTT = oral glucose tolerance test
