# Supplementary material for: The association of red and processed meat with gestational diabetes mellitus: Results from 2 Canadian birth cohort studies
Source: PLoS One. 2024 May 30;19(5):e0302208. doi: 10.1371/journal.pone.0302208 (PMC11139301; doi:10.1371/journal.pone.0302208)
Supplement: S1 Table — (DOCX) [file pone.0302208.s002.docx]

S2 Table. Listing of Red Meat and Processed Meat Classification by Cohorts

| **Food Group** | **FAMILY Food Items** | **START Food Items** |
| --- | --- | --- |
| Red Meat | Ground beef  Roast beef  Steak  Pot roast  Pork chop  Baked ham  Veal  Lamb  Lunch ham /corned beef | Roast beef /steak  Pork chop /mince /keema /kabob /dry kofta  Ground beef /mince /hamburger /keema kabob /dry kofta  Goat /lamb mince /roast /steak /chop /keema /kabob /dry kofta /raan  Beef curry /kofta  Pork curry /kofta  Goat /lamb curry |
| Likely Red Meat, Processed  (80% RM unless otherwise specified) | Hot dog  Sausage  Other lunch meat  Bacon  Pickled meat /fish (50%)*  Salted /dried meat /fish (50%)* | Hot dog /sausage  Lunch meat |

*We assumed that 50% of these two categories were servings of red meat and were counted as such.
