# Supplementary material for: The association of red and processed meat with gestational diabetes mellitus: Results from 2 Canadian birth cohort studies
Source: PLoS One. 2024 May 30;19(5):e0302208. doi: 10.1371/journal.pone.0302208 (PMC11139301; doi:10.1371/journal.pone.0302208)
Supplement: S2 Table — (DOCX) [file pone.0302208.s003.docx]

S3 Table. Participant characteristics according to categories of dietary intake of red meat and processed red meat among 581 expectant mothers in the FAMILY cohort and 976 expectant mothers in the START cohort

| Variables | FAMILY (n = 581) | | | | | | | | | | | | | | START (n = 976) | | | | | | | | | | | | | | | |
| --- | --- | --- | --- | --- | --- | --- | --- | --- | --- | --- | --- | --- | --- | --- | --- | --- | --- | --- | --- | --- | --- | --- | --- | --- | --- | --- | --- | --- | --- | --- |
|  | Unprocessed Red Meat | | | | | | | Processed Meat | | | | | | | Unprocessed Red Meat | | | | | | |  |  | Processed Meat | | | | | | |
|  | Low |  | Med |  | High |  | p-trend+ | Low |  | Med |  | High |  | p-trend+ | Low | | Med |  | High |  | p-trend+ | NC |  | Low |  | Med |  | High |  | p-trend+ |
|  | **n** | **%** | **n** | **%** | **n** | **%** |  | **n** | **%** | **n** | **%** | **n** | **%** |  | **n** | **%** | **n** | **%** | **n** | **%** |  | **n** | **%** | **n** | **%** | **n** | **%** | **n** | **%** |  |
| Participants | **193** |  | **194** |  | **194** |  |  | **193** |  | **194** |  | **194** |  |  | **325** |  | **326** |  | **325** |  |  | **565** |  | **139** |  | **135** |  | **137** |  |  |
| Median (g/d) | **20.5** | | **44.1** | | **75.6** | |  | **4.7** | | **11.5** | | **24.3** | |  | **0.06** | | **1.3** | | **19.7** | |  | **0.00** | | **0.02** | | **0.22** | | **1.4** | |  |
| Maternal Age (M/SD) | 31.8 | (4.2) | 31.3 | (4.7) | 31.1 | (4.7) | 0.14 | 32.0 | (4.2) | 31.5 | (4.5) | 30.8 | (4.8) | 0.01 | 30.0 | (3.9) | 30.5 | (3.7) | 30.0 | (4.1) | 0.85 | 29.9 | (3.9) | 30.8 | (4.0) | 30.1 | (3.8) | 30.5 | (3.8) | 0.10 |
| Pre-pregnancy BMI (kg/m^2) (M/SD) | 25.7 | (5.5) | 26.0 | (5.9) | 27.3 | (7.1) | 0.01 | 25.0 | (5.0) | 27.0 | (6.5) | 27.0 | (6.9) | 0.001 | 23.4 | (4.2) | 23.6 | (4.5) | 24.4 | (4.8) | 0.01 | 23.6 | (4.4) | 23.6 | (4.4) | 24.5 | (5.1) | 24.4 | (4.7) | 0.02 |
| Gestational weight gain (kg) (M/SD) | 14.8 | (5.3) | 15.3 | (4.9) | 14.6 | (5.4) | 0.75 | 14.4 | (5.2) | 15.0 | (5.2) | 15.2 | (5.2) | 0.14 | 14.7 | (9.6) | 14.5 | (7.7) | 13.8 | (5.6) | 0.13 | 14.4 | (8.2) | 14.5 | (6.0) | 14.9 | (9.4) | 13.3 | (5.3) | 0.37 |
| Maternal Family Hx of DM | 41 | 22.4 | 37 | 21.0 | 43 | 24.6 | 0.63 | 36 | 19.9 | 44 | 24.6 | 41 | 23.6 | 0.4 | 113 | 35.1 | 139 | 43.6 | 163 | 50.8 | <0.01 | 227 | 40.7 | 56 | 40.9 | 62 | 47.3 | 70 | 51.5 |  |
| Parity (median/IQR) | 1 | (1-4) | 1 | (1-4) | 1 | (1-4) | 0.18 | 0 | (1-4) | 1 | (1-4) | 1 | (1-4) | 0.04 | 1 | (1-4) | 1 | (1-3) | 1 | (2-4) | <0.001 | 1 | (1-4) | 1 | (1-4) | 1 | (1-4) | 1 | (1-4) | 0.11 |
| Marital status |  |  |  |  |  |  | 0.32 |  |  |  |  |  |  | 0.05 |  |  |  |  |  |  | - |  |  |  |  |  |  |  |  | - |
| Married or Common Law | 160 | 95.8 | 164 | 93.2 | 148 | 92.5 |  | 164 | 97.0 | 158 | 93.5 | 150 | 90.9 |  | 324 | 100.0 | 326 | 100.0 | 325 | 100.0 |  | 564 | 100.0 | 139 | 100.0 | 135 | 100.0 | 137 | 100.0 |  |
| Never Married | 3 | 1.8 | 8 | 4.6 | 10 | 6.3 |  | 1 | 0.6 | 9 | 5.3 | 11 | 2.4 |  | - | - |  | - | - | - |  | - | - | - | - | - | - | - | - |  |
| Divorced or Separated | 4 | 2.4 | 4 | 2.3 | 2 | 1.3 |  | 4 | 2.4 | 2 | 1.2 | 4 | 2.4 |  | - | - |  | - | - | - |  | - | - | - | - | - | - | - | - |  |
| Smoking Hx |  |  |  |  |  |  | 0.20 |  |  |  |  |  |  | 0.10 |  |  |  |  |  |  | 0.09 |  |  |  |  |  |  |  |  | 0.56 |
| Never smoked | 121 | 64.0 | 125 | 65.5 | 114 | 60 |  | 128 | 67.7 | 123 | 65.1 | 109 | 56.8 |  | 324 | 100 | 326 | 100 | 319 | 98.8 |  | 561 | 99.6 | 139 | 100 | 133 | 99.3 | 136 | 99.3 |  |
| Quit before this pregnancy | 34 | 18.0 | 23 | 12 | 31 | 16.3 |  | 30 | 15.9 | 28 | 14.8 | 30 | 15.6 |  | 0 | 0 | 0 | 0 | 2 | 0.6 |  | 1 | 0.2 | 0 | 0.0 | 1 | 0.8 | 0 | 0.0 |  |
| Quit during this pregnancy | 27 | 14.3 | 35 | 18.3 | 29 | 15.3 |  | 26 | 13.8 | 29 | 15.3 | 36 | 18.8 |  | 0 | 0 | 0 | 0 | 2 | 0.6 |  | 1 | 0.2 | 0 | 0.0 | 0 | 0.0 | 1 | 0.7 |  |
| Currently smoking | 7 | 3.7 | 8 | 4.2 | 16 | 8.4 |  | 5 | 2.7 | 9 | 4.8 | 17 | 8.9 |  | - | - | - | - | - | - |  | - | - | - | - | - | - | - | - |  |
| Employment status |  |  |  |  |  |  | 0.01 |  |  |  |  |  |  | 0.01 |  |  |  |  |  |  | <0.01 |  |  |  |  |  |  |  |  | 0.17 |
| Unemployed or retired | 23 | 11.9 | 29 | 15 | 43 | 22.2 |  | 22 | 11.4 | 28 | 14.4 | 45 | 23.2 |  | 143 | 44.4 | 109 | 33.5 | 192 | 59.1 |  | 260 | 46.4 | 60 | 43.2 | 72 | 53.3 | 52 | 38.0 |  |
| Employed part-time | 32 | 16.6 | 45 | 23.2 | 44 | 22.7 |  | 39 | 20.2 | 37 | 19.1 | 45 | 23.2 |  | 30 | 9.3 | 31 | 9.5 | 27 | 8.3 |  | 49 | 8.7 | 13 | 9.4 | 8 | 5.9 | 18 | 13.1 |  |
| Employed full-time | 138 | 71.5 | 120 | 61.9 | 107 | 55.2 |  | 132 | 68.4 | 129 | 66.5 | 104 | 53.6 |  | 149 | 46.3 | 185 | 56.9 | 106 | 32.6 |  | 252 | 44.9 | 66 | 47.5 | 55 | 40.7 | 67 | 49 |  |
| Annual household income |  |  |  |  |  |  | <0.001 |  |  |  |  |  |  | <0.001 |  |  |  |  |  |  | 0.01 |  |  |  |  |  |  |  |  | <0.001 |
| <$30K | 12 | 6.4 | 7 | 3.7 | 25 | 13.1 |  | 7 | 3.7 | 11 | 5.8 | 26 | 13.6 |  | 84 | 30.1 | 63 | 22.5 | 74 | 26.3 |  | 145 | 30.3 | 30 | 24 | 31 | 26.1 | 15 | 12.7 |  |
| $30K-49 999K | 12 | 6.4 | 27 | 14.1 | 27 | 14.1 |  | 21 | 11.1 | 18 | 9.4 | 27 | 14.1 |  | 94 | 33.7 | 82 | 29.3 | 71 | 25.3 |  | 149 | 31.2 | 38 | 30..4 | 36 | 30.2 | 24 | 20.3 |  |
| >$50K | 165 | 87.3 | 157 | 82.2 | 139 | 72.8 |  | 161 | 85.2 | 162 | 84.8 | 138 | 72.3 |  | 101 | 36.2 | 135 | 48.2 | 136 | 48.4 |  | 184 | 38.5 | 57 | 45.6 | 52 | 43.7 | 79 | 67 |  |
| Mom completed high school | 190 | 98.5 | 190 | 97.9 | 189 | 97.4 | 0.48 | 190 | 98.5 | 192 | 99.0 | 187 | 96.4 | 0.15 | 323 | 99.7 | 325 | 99.7 | 323 | 99.4 | 0.54 | 560 | 99.3 | 139 | 100 | 135 | 100 | 137 | 100 | 0.13 |
| Hours of active sport/week during pregnancy (median/IQR) | 2 | (0-4) | 1 | (0-3) | 1 | (0-4) | 0.16 | 2 | (0-4) | 1 | (0-4) | 0 | (0-3) | <0.001 | 0 | (0-4) | 0 | (0-4) | 0 | (0-2) | <0.001 | 0 | (0-4) | 0 | (0-2) | 0 | (0-2) | 0 | (0-2) | 0.01 |
| Social Disadvantage index |  |  |  |  |  |  | 0.06 |  |  |  |  |  |  | <0.001 |  |  |  |  |  |  | <0.001 |  |  |  |  |  |  |  |  | <0.01 |
| Low | 142 | 87.1 | 149 | 85.6 | 120 | 76 |  | 145 | 87.4 | 144 | 86.8 | 122 | 74.9 |  | 116 | 42.0 | 156 | 55.7 | 110 | 39.2 |  | 197 | 41.5 | 61 | 48.8 | 50 | 42.0 | 74 | 62.7 |  |
| Moderate | 15 | 9.2 | 21 | 12.1 | 30 | 19 |  | 18 | 10.8 | 19 | 11.5 | 29 | 17.8 |  | 121 | 43.8 | 91 | 32.5 | 111 | 39.5 |  | 199 | 41.9 | 45 | 36.0 | 47 | 39.5 | 32 | 27.1 |  |
| High | 6 | 3.7 | 4 | 2.3 | 8 | 5.1 |  | 3 | 1.8 | 3 | 1.8 | 12 | 7.4 |  | 39 | 14.1 | 33 | 11.8 | 60 | 21.4 |  | 79 | 16.6 | 19 | 15.2 | 22 | 18.5 | 12 | 10.2 |  |
| GDM | 29 | 15.0 | 27 | 13.9 | 35 | 18.0 | 0.41 | 21 | 10.9 | 32 | 16.5 | 38 | 19.6 | 0.02 | 75 | 23.1 | 81 | 24.9 | 85 | 26.2 | 0.36 | 129 | 22.8 | 45 | 32.4 | 32 | 23.7 | 35 | 25.6 | 0.44 |
| GDM by CDA | 7 | 3.8 | 5 | 2.7 | 2 | 1.1 | 0.1 | 3 | 1.6 | 3 | 1.6 | 8 | 4.3 | 0.09 | 20 | 6.6 | 22 | 7.2 | 28 | 9.1 | 0.24 | 34 | 6.4 | 18 | 13.9 | 8 | 6.4 | 10 | 7.8 | 0.54 |
| GDM by IADPSG | 28 | 15.1 | 24 | 12.8 | 31 | 16.7 | 0.66 | 20 | 10.6 | 29 | 15.7 | 34 | 18.3 | 0.04 | 67 | 22 | 70 | 23 | 72 | 23.5 | 0.68 | 113 | 21.2 | 42 | 32.3 | 26 | 20.6 | 28 | 21.9 | 0.82 |
| GDM by BiB | - | - | - | - | - | - | - | - | - | - | - | - | - | - | 115 | 35.4 | 112 | 34.4 | 126 | 38.9 | 0.37 | 196 | 34.7 | 60 | 43.2 | 48 | 35.6 | 49 | 35.8 | 0.7 |

GDM - The mother reports having GDM or using insulin during pregnancy, it is specified on her birth chart, or her OGTT came out positive, using IADPSG thresholds

GDM by CDA - The mother meets or exceeds at least two CDA-defined OGTT thresholds

GDM by IADPSG - The mother meets or exceeds any of the OGTT thresholds defined by the IADPSG: Base = 5.1; 1 hour = 10.0; 2 hour = 8.5

GDM by BiB - The mother reports having GDM or using insulin during pregnancy, it is specified on her birth chart, or her OGTT came out positive, using Born-in-Bradford thresholds

^†^P-trend were calculated with the use of linear regression, Jonckheere-Terpstra, Cochran-Armitage or Cochran Mantel-Haenszel tests, where appropriate

**+Data are presented as mean ± SD, median (interquartile range) or percentage, where appropriate. Percentages are rounded to one decimal place
