# Supplementary material for: The association of red and processed meat with gestational diabetes mellitus: Results from 2 Canadian birth cohort studies
Source: PLoS One. 2024 May 30;19(5):e0302208. doi: 10.1371/journal.pone.0302208 (PMC11139301; doi:10.1371/journal.pone.0302208)
Supplement: S3 Table — A. Association between low and high red and processed meat intake relative to medium intake and gestational diabetes mellitus (GDM)–CDA. B. Association between low and high red and processed meat intake relative to medium intake and gestational diabetes mellitus (GDM)–IADPSG. C. Association between low and high red and processed meat intake relative to medium intake and gestational diabetes mellitus (GDM)–Born-in-Bradford. (DOCX) [file pone.0302208.s004.docx]

S4A Table. Association between low and high red and processed meat intake relative to medium intake and gestational diabetes mellitus (GDM) - CDA

| **Variable** | **FAMILY (n = 559)** | | | | | |  |  | **START (n = 916)** | | | | | | |
| --- | --- | --- | --- | --- | --- | --- | --- | --- | --- | --- | --- | --- | --- | --- | --- |
|  | **Low** |  | **Medium** | **High** |  | **p-trend** | **NC** |  | **Low** |  | **Medium** | **High** |  | **p-trend** |  |
|  | **OR** | **95% CI** |  | **OR** | **95% CI** |  | **OR** | **95% CI** | **OR** | **95% CI** |  | **OR** | **95% CI** |  |  |
| **Unprocessed Red Meat median (g/d)** | **20.5** | | **44.1** | **75.8** | |  |  | | **0.06** | | **1.3** | **19.3** | |  | |
| **GDM cases/ pregnancies** | 7/186 | | 5/187 | 2/186 | |  |  |  | 20/304 | | 22/305 | 28/307 | |  | |
| Unadjusted | 1.42 | 0.44 - 4.57 | 1 (Ref.) | 0.4 | 0.08 - 2.07 | 0.11 | - | - | 0.91 | 0.48 - 1.70 | 1 (Ref.) | 1.29 | 0.72 - 2.31 | 0.23 |  |
| Model 1 | 1.43 | 0.44 - 4.60 | 1 (Ref.) | 0.4 | 0.08 - 2.11 | 0.11 | - | - | 0.95 | 0.50 - 1.80 | 1 (Ref.) | 1.47 | 0.81 - 2.66 | 0.12 |  |
| Model 2 | 1.27 | 0.36 - 4.50 | 1 (Ref.) | 0.27 | 0.05 - 1.57 | 0.08 | - | - | 1.01 | 0.53 - 1.94 | 1 (Ref.) | 1.5 | 0.81 - 2.77 | 0.14 |  |
| Model 3 | 0.63 | 0.14 - 2.80 | 1 (Ref.) | 0.4 | 0.06 - 2.57 | 0.71 | - | - | 1.06 | 0.55 - 2.04 | 1 (Ref.) | 1.33 | 0.72 - 2.48 | 0.35 |  |
| Model 4 | 0.66 | 0.14 - 3.09 | 1 (Ref.) | 0.34 | 0.05 - 2.50 | 0.61 | - | - | 0.97 | 0.50 - 1.90 | 1 (Ref.) | 1.5 | 0.79 - 2.86 | 0.15 |  |
| ***Processed Meat median (g/d)** | **4.7** | | **11.5** | **24.3** | |  | **0.00** | | **0.02** | | **0.22** | **1.4** | |  | |
| **GDM cases/ pregnancies** | 3/188 | | 3/185 | 8/186 | |  | 34/532 | | 18/130 | | 8/126 | 10/128 | |  | |
| Unadjusted | 0.99 | 0.20 - 4.94 | 1 (Ref.) | 2.73 | 0.71 - 10.44 | 0.08 | 1.01 | 0.45 - 2.23 | 2.37 | 0.99 - 5.67 | 1 (Ref.) | 1.25 | 0.48 - 3.28 | 0.97 |  |
| Model 1 | 0.98 | 0.19 - 4.94 | 1 (Ref.) | 2.85 | 0.74 - 11.0 | 0.07 | 0.97 | 0.43 - 2.18 | 2.03 | 0.83 - 4.94 | 1 (Ref.) | 1.15 | 0.43 - 3.05 | 0.97 |  |
| Model 2 | 2.09 | 0.32 - 13.90 | 1 (Ref.) | 4.88 | 0.93 - 25.49 | 0.09 | 1.04 | 0.45 - 2.39 | 2.49 | 0.99 - 6.23 | 1 (Ref.) | 1.05 | 0.39 - 2.88 | 0.62 |  |
| Model 3 | 2.16 | 0.29 - 16.16 | 1 (Ref.) | 8.57 | 1.21 - 60.58 | 0.05 | 1.03 | 0.45 - 2.39 | 2.42 | 0.96 - 6.15 | 1 (Ref.) | 1.00 | 0.36 - 2.74 | 0.49 |  |
| Model 4 | 2.39 | 0.30 - 19.14 | 1 (Ref.) | 8.98 | 1.25 - 64.36 | 0.05 | 0.92 | 0.39 - 2.17 | 2.39 | 0.93 - 6.10 | 1 (Ref.) | 1.08 | 0.39 - 2.98 | 0.75 |  |
| **Total Red and Processed Meat median (g/d)** | **30.2** | | **58.2** | **96.8** | |  | **-** | - | **0.06** | | **1.5** | **20.5** | |  | |
| **GDM cases/ pregnancies** | 5/187 | | 6/187 | 3/185 | |  | - | - | 20/305 | | 22/304 | 28/307 | |  | |
| Unadjusted | 0.83 | 0.25 - 2.76 | 1 (Ref.) | 0.50 | 1.22 - 2.02 | 0.48 | - | - | 0.90 | 0.48 - 1.69 | 1 (Ref.) | 1.29 | 0.72 - 2.30 | 0.23 |  |
| Model 1 | 0.84 | 0.25 - 2.81 | 1 (Ref.) | 0.51 | 0.12 - 2.06 | 0.48 | - | - | 0.96 | 0.51 - 1.82 | 1 (Ref.) | 1.49 | 0.82 - 2.71 | 0.11 |  |
| Model 2 | 0.73 | 0.19 - 2.77 | 1 (Ref.) | 0.39 | 0.01 - 1.75 | 0.41 | - | - | 1.05 | 0.55 - 2.02 | 1 (Ref.) | 1.56 | 0.85 - 2.89 | 0.12 |  |
| Model 3 | 0.40 | 0.08 - 2.03 | 1 (Ref.) | 0.82 | 0.16 - 4.28 | 0.52 | - | - | 1.10 | 0.57 - 2.13 | 1 (Ref.) | 1.42 | 0.76 - 2.64 | 0.28 |  |
| Model 4 | 0.39 | 0.07 - 2.11 | 1 (Ref.) | 0.77 | 0.14 - 4.15 | 0.58 | - | - | 1.00 | 0.51 - 1.96 | 1 (Ref.) | 1.61 | 0.84 - 3.07 | 0.11 |  |

Model 1: Adjusted for age and parity

Model 2: Adjusted for age, parity, pre-pregnancy BMI, pregnancy weight gain

Model 3: Adjusted for age, parity, pre-pregnancy BMI, pregnancy weight gain, smoking (FAMILY only), family history of DM, level of education, total energy

Model 4: Adjusted for age, parity, pre-pregnancy BMI, pregnancy weight gain, smoking (FAMILY only), family history of DM, level of education, total energy, diet quality score, total fiber, saturated fat and glycemic load

*Processed Meat for START cohort is grouped based on non-consumers (NC), low, medium and high consumers

S4B Table. Association between low and high red and processed meat intake relative to medium intake and gestational diabetes mellitus (GDM) - IADPSG

| **Variable** | **FAMILY (n = 559)** | | | | | | **START (n = 916)** | | | | | | | | |
| --- | --- | --- | --- | --- | --- | --- | --- | --- | --- | --- | --- | --- | --- | --- | --- |
|  | **Low** |  | **Medium** | **High** |  | **p-trend** | **NC** |  | **Low** |  | **Medium** | **High** |  | **p-trend** |  |
|  | **OR** | **95% CI** |  | **OR** | **95% CI** |  | **OR** | **95% CI** | **OR** | **95% CI** |  | **OR** | **95% CI** |  |  |
| **Unprocessed Red Meat median (g/d)** | **20.5** | | **44.1** | **75.68** | |  |  | | **0.06** | | **1.3** | **19.3** | |  |  |
| **GDM cases/ pregnancies** | 28/186 | | 24/187 | 31/186 | |  |  |  | 67/304 | | 70/305 | 72/307 | |  |  |
| Unadjusted | 1.20 | 0.67 - 2.17 | 1 (Ref.) | 1.36 | 0.76 - 2.42 | 0.61 | - | - | 0.95 | 0.65 - 1.39 | 1 (Ref.) | 1.03 | 0.71 - 1.50 | 0.73 |  |
| Model 1 | 1.18 | 0.66 - 2.14 | 1 (Ref.) | 1.39 | 0.78 - 2.49 | 0.52 | - | - | 1.00 | 0.68 - 1.47 | 1 (Ref.) | 1.09 | 0.74 - 1.61 | 0.61 |  |
| Model 2 | 1.20 | 0.62 - 2.30 | 1 (Ref.) | 1.18 | 0.62 - 2.25 | 1 | - | - | 1.04 | 0.70 - 1.55 | 1 (Ref.) | 1.10 | 0.74 - 1.63 | 0.68 |  |
| Model 3 | 1.14 | 0.55 - 2.34 | 1 (Ref.) | 1.25 | 0.60 - 2.63 | 0.79 | - | - | 1.08 | 0.72 - 1.61 | 1 (Ref.) | 1.00 | 0.67 - 1.50 | 0.84 |  |
| Model 4 | 1.72 | 0.57 - 2.43 | 1 (Ref.) | 1.21 | 0.56 - 2.57 | 0.93 | - | - | 1.05 | 0.70 - 1.58 | 1 (Ref.) | 1.04 | 0.68 - 1.58 | 0.95 |  |
| ***Processed Meat median (g/d)** | **4.7** | | **11.5** | **24.3** | |  | **0.0** | | **0.02** | | **0.2** | **1.4** | |  |  |
| **GDM cases/ pregnancies** | 20/188 | | 29/185 | 34/186 | |  | 113/532 | | 42/130 | | 26/126 | 28/128 | |  |  |
| Unadjusted | 0.64 | 0.35 - 1.18 | 1 (Ref.) | 1.2 | 0.70 - 2.07 | 0.05 | 1.04 | 0.64 - 1.67 | 1.84 | 1.04 - 3.24 | 1 (Ref.) | 1.08 | 0.60 - 1.97 | 0.74 |  |
| Model 1 | 0.62 | 0.34 - 1.15 | 1 (Ref.) | 1.26 | 0.73 - 2.19 | 0.03 | 1.05 | 0.64 - 1.71 | 1.69 | 0.95 - 3.02 | 1 (Ref.) | 1.03 | 0.56 - 1.90 | 0.63 |  |
| Model 2 | 0.87 | 0.44 - 1.72 | 1 (Ref.) | 1.51 | 0.81 - 2.82 | 0.08 | 1.08 | 0.65 - 1.78 | 1.77 | 0.98 - 3.23 | 1 (Ref.) | 0.94 | 0.50 - 1.77 | 0.35 |  |
| Model 3 | 0.93 | 0.45 - 1.91 | 1 (Ref.) | 1.54 | 0.76 - 3.12 | 0.16 | 1.04 | 0.62 - 1.73 | 1.77 | 0.97 - 3.24 | 1 (Ref.) | 0.91 | 0.48 - 1.71 | 0.32 |  |
| Model 4 | 0.96 | 0.46 - 2.02 | 1 (Ref.) | 1.52 | 0.74 - 3.10 | 0.21 | 0.98 | 0.58 - 1.66 | 1.77 | 0.96 - 3.26 | 1 (Ref.) | 0.93 | 0.49 - 1.77 | 0.48 |  |
| **Total Red and Processed Meat median (g/d)** | **30.1** | | **58.2** | **96.8** | |  | **-** | - | **0.06** | | **1.5** | **20.5** | |  |  |
| **GDM cases/ pregnancies** | 23/187 | | 29/187 | 31/185 | |  | - | - | 67/305 | | 69/304 | 73/307 | |  |  |
| Unadjusted | 0.76 | 0.42 - 1.38 | 1 (Ref.) | 1.1 | 0.63 - 1.91 | 0.24 | - | - | 0.96 | 0.66 - 1.40 | 1 (Ref.) | 1.06 | 0.73 -1 .54 | 0.61 |  |
| Model 1 | 0.76 | 0.42 - 1.38 | 1 (Ref.) | 1.14 | 0.65 - 2.00 | 0.19 | - | - | 1.02 | 0.69 - 1.51 | 1 (Ref.) | 1.15 | 0.78 - 1.69 | 0.47 |  |
| Model 2 | 0.74 | 0.38 - 1.42 | 1 (Ref.) | 0.96 | 0.52 - 1.79 | 0.49 | - | - | 1.08 | 0.72 - 1.60 | 1 (Ref.) | 1.16 | 0.78 - 1.73 | 0.53 |  |
| Model 3 | 0.72 | 0.35 - 1.49 | 1 (Ref.) | 1.17 | 0.57 - 2.40 | 0.27 | - | - | 1.12 | 0.75 - 1.68 | 1 (Ref.) | 1.08 | 0.72 - 1.62 | 0.91 |  |
| Model 4 | 0.75 | 0.36 - 1.58 | 1 (Ref.) | 1.15 | 0.56 - 2.38 | 0.35 | - | - | 1.09 | 0.72 - 1.64 | 1 (Ref.) | 1.13 | 0.74 - 1.72 | 0.68 |  |

Model 1: Adjusted for age and parity

Model 2: Adjusted for age, parity, pre-pregnancy BMI, pregnancy weight gain

Model 3: Adjusted for age, parity, pre-pregnancy BMI, pregnancy weight gain, smoking (FAMILY only), family history of DM, level of education, total energy

Model 4: Adjusted for age, parity, pre-pregnancy BMI, pregnancy weight gain, smoking (FAMILY only), family history of DM, level of education, total energy, diet quality score, total fiber, saturated fat and glycemic load

*Processed Meat for START cohort is grouped based on non-consumers (NC), low, medium and high consumers

S4C Table. Association between low and high red and processed meat intake relative to medium intake and gestational diabetes mellitus (GDM) – Born-in-Bradford

| Variable | START (n = 976) | | | | | | | |
| --- | --- | --- | --- | --- | --- | --- | --- | --- |
|  | **NC** |  | **Low** |  | **Medium** | **High** |  | **p-trend** |
|  | **OR** | **95% CI** | **OR** | **95% CI** |  | **OR** | **95% CI** |  |
| Unprocessed Red Meat median (g/d) | **-** | | **0.06** | | **1.3** | **19.7** | |  |
| GDM cases/ pregnancies | - | | 67/304 | | 70/305 | 72/307 | |  |
| Unadjusted | - | - | 1.05 | 0.76 – 1.44 | 1 (Ref.) | 1.21 | 0.88 – 1.67 | 0.24 |
| Model 1 | - | - | 1.10 | 0.79 – 1.53 | 1 (Ref.) | 1.26 | 0.91 – 1.76 | 0.22 |
| Model 2 | - | - | 1.13 | 0.81 – 1.59 | 1 (Ref.) | 1.30 | 0.93 – 1.83 | 0.19 |
| Model 3 | - | - | 1.13 | 0.80 – 1.59 | 1 (Ref.) | 1.20 | 0.85 – 1.70 | 0.44 |
| Model 4 | - | - | 1.09 | 0.77 – 1.55 | 1 (Ref.) | 1.25 | 0.87 – 1.79 | 0.29 |
| *Processed Meat median (g/d) | **0.0** | | **0.02** | | **0.2** | **1.4** | |  |
| GDM cases/ pregnancies | 196/565 | | 60/139 | | 48/135 | 49/137 | |  |
| Unadjusted | 0.96 | 0.65 – 1.43 | 1.38 | 0.85 – 2.24 | 1 (Ref.) | 1.01 | 0.61 – 1.66 | 0.92 |
| Model 1 | 0.99 | 0.66 – 1.47 | 1.30 | 0.79 – 2.15 | 1 (Ref.) | 0.97 | 0.59 – 1.61 | 0.73 |
| Model 2 | 0.97 | 0.64 – 1.47 | 1.33 | 0.79 – 2.21 | 1 (Ref.) | 0.88 | 0.52 – 1.47 | 0.42 |
| Model 3 | 0.96 | 0.63 – 1.46 | 1.32 | 0.78 – 2.22 | 1 (Ref.) | 0.84 | 0.49 – 1.43 | 0.35 |
| Model 4 | 0.90 | 0.58 – 1.39 | 1.30 | 0.77 – 2.20 | 1 (Ref.) | 0.85 | 0.50 – 1.45 | 0.54 |
| Total Red and Processed Meat median (g/d) | **-** | - | **0.06** | | **1.5** | **20.5** | |  |
| GDM cases/ pregnancies | - | - | 67/305 | | 69/304 | 73/307 | |  |
| Unadjusted | - | - | 1.03 | 0.75 – 1.42 | 1 (Ref.) | 1.18 | 0.86 – 1.62 | 0.30 |
| Model 1 | - | - | 1.10 | 0.79 – 1.52 | 1 (Ref.) | 1.24 | 0.89 – 1.73 | 0.26 |
| Model 2 | - | - | 1.13 | 0.80 – 1.56 | 1 (Ref.) | 1.28 | 0.91 – 1.80 | 0.22 |
| Model 3 | - | - | 1.13 | 0.80 – 1.59 | 1 (Ref.) | 1.20 | 0.85 – 1.69 | 0.46 |
| Model 4 | - | - | 1.10 | 0.77 – 1.56 | 1 (Ref.) | 1.26 | 0.90 – 1.81 | 0.26 |

Model 1: Adjusted for age and parity

Model 2: Adjusted for age, parity, pre-pregnancy BMI, pregnancy weight gain

Model 3: Adjusted for age, parity, pre-pregnancy BMI, pregnancy weight gain, family history of DM, level of education, total energy

Model 4: Adjusted for age, parity, pre-pregnancy BMI, pregnancy weight gain, family history of DM, level of education, total energy, diet quality score, total fiber, saturated fat and glycemic load

*Processed Meat is grouped based on non-consumers (NC), low, medium and high consumers
