# Supplementary material for: The association of red and processed meat with gestational diabetes mellitus: Results from 2 Canadian birth cohort studies
Source: PLoS One. 2024 May 30;19(5):e0302208. doi: 10.1371/journal.pone.0302208 (PMC11139301; doi:10.1371/journal.pone.0302208)
Supplement: S4 Table — (DOCX) [file pone.0302208.s005.docx]

S5 Table. Odds Risk for gestational diabetes mellitus (GDM) according to 90 grams of red and processed meat intake by cohort

| Variable | FAMILY (n = 581) | | | START (n = 976) | | |
| --- | --- | --- | --- | --- | --- | --- |
|  | **per 90g increase** | | **p-value** | **per 90g increase** | | **p-value** |
|  | **OR** | **95% CI** |  | **OR** | **95% CI** |  |
| Unprocessed Red Meat median (IQR) (g/d) | **44.1** | **(27.5 – 65.9)** |  | **1.3** | **(0.2 – 11.0)** |  |
| GDM cases/ pregnancies | 91/581 | |  | 241/976 | |  |
| Unadjusted | 1.16 | 0.60 - 2.24 | 0.66 | 1.34 | 0.61 - 2.94 | 0.46 |
| Model 1 | 1.21 | 0.62 - 2.37 | 0.58 | 1.55 | 0.68 - 3.49 | 0.30 |
| Model 2 | 0.95 | 0.45 - 1.99 | 0.89 | 1.59 | 0.69 - 3.64 | 0.27 |
| Model 3 | 1.04 | 0.40 - 2.74 | 0.94 | 1.51 | 0.63 - 3.59 | 0.35 |
| Model 4 | 0.90 | 0.33 - 2.50 | 0.85 | 1.70 | 0.66 - 4.37 | 0.30 |
| *Processed Meat median (IQR) (g/d) | **11.5** | **(6.2 – 19.7)** |  | **-** | **-** | **-** |
| GDM cases/ pregnancies | 91/581 | |  |  | |  |
| Unadjusted | 2.68 | 0.61 - 11.77 | 0.19 | - | - | - |
| Model 1 | 3.25 | 0.72 - 14.65 | 0.13 | - | - | - |
| Model 2 | 1.65 | 0.30 - 9.17 | 0.57 | - | - | - |
| Model 3 | 2.25 | 0.30 - 16.97 | 0.43 | - | - | - |
| Model 4 | 1.72 | 0.20 - 14.62 | 0.62 | - | - | - |
| Total Red and Processed Meat median (IQR) (g/d) | **58.2** | **(37.1 – 85.7)** |  | **1.5** | **(0.2 – 11.3)** |  |
| GDM cases/ pregnancies | 91/581 | |  | 241/976 | |  |
| Unadjusted | 1.24 | 0.73 - 2.13) | 0.42 | 1.36 | 0.63 - 2.92 | 0.43 |
| Model 1 | 1.32 | 0.76 - 2.28) | 0.33 | 1.54 | 0.70 - 3.40 | 0.29 |
| Model 2 | 1.03 | 0.56 - 1.89) | 0.94 | 1.55 | 0.69 - 3.49 | 0.28 |
| Model 3 | 1.16 | 0.52 - 2.63) | 0.71 | 1.50 | 0.65 - 3.50 | 0.35 |
| Model 4 | 1.01 | 0.42 - 2.42) | 0.98 | 1.70 | 0.68 - 4.27 | 0.26 |

Model 1: Adjusted for age and parity

Model 2: Adjusted for age, parity, pre-pregnancy BMI, pregnancy weight gain

Model 3: Adjusted for age, parity, pre-pregnancy BMI, pregnancy weight gain, smoking (FAMILY only), family history of DM, level of education, total energy

Model 4: Adjusted for age, parity, pre-pregnancy BMI, pregnancy weight gain, smoking (FAMILY only), family history of DM, level of education, total energy, diet quality, total fiber, saturated fat and glycemic load

*Processed Meat for START cohort is grouped based on non-consumers (NC), low, medium and high consumers

For processed red meat in the START cohort are not present due to low power to calculate estimates
