# Supplementary material for: The association of red and processed meat with gestational diabetes mellitus: Results from 2 Canadian birth cohort studies
Source: PLoS One. 2024 May 30;19(5):e0302208. doi: 10.1371/journal.pone.0302208 (PMC11139301; doi:10.1371/journal.pone.0302208)
Supplement: S5 Table — A. Odds Ratio (95% confidence interval) for gestational diabetes mellitus (GDM) according to red and processed meat intake stratified by BMI–FAMILY cohort. B. Odds Ratio (95% confidence interval) for gestational diabetes mellitus (GDM) according to red and processed meat intake stratified by BMI–START cohort. (DOCX) [file pone.0302208.s006.docx]

S6A Table. Odds Ratio (95% confidence interval) for gestational diabetes mellitus (GDM) according to red and processed meat intake stratified by BMI – FAMILY cohort

| Meat | FAMILY (n = 581) | | | | | | | | | |
| --- | --- | --- | --- | --- | --- | --- | --- | --- | --- | --- |
|  | **BMI <25** | | | |  | **BMI >25** | | | | |
|  | **Low** | | **Medium** | **High** | | **Low** | | **Medium** | **High** | |
|  | **OR** | **95% CI** |  | **OR** | **95% CI** | **OR** | **95% CI** |  | **OR** | **95% CI** |
| Unprocessed Red Meat | 1.67 | 0.49 - 5.77 | 1 (Ref.) | 2.00 | 0.52 - 7.66 | 0.74 | 0.31 - 1.75 | 1 (Ref.) | 1.12 | 0.48 - 2.57 |
| Processed Meat | 0.52 | 0.16 - 1.77 | 1 (Ref.) | 0.55 | 0.16 - 1.88 | 1.02 | 0.44 - 2.37 | 1 (Ref.) | 2.21 | 0.98 - 5.01 |
| Total Red and Processed Meat | 0.77 | 0.22 - 2.67 | 1 (Ref.) | 1.44 | 0.42 - 5.00 | 0.65 | 0.27 - 1.56 | 1 (Ref.) | 1.32 | 0.58 - 2.98 |

Model adjusted for age, parity, pre-pregnancy BMI, pregnancy weight gain, smoking, family history of DM, level of education, total energy, diet quality score, total fiber, saturated fat and glycemic load

S6B Table. Odds Ratio (95% confidence interval) for gestational diabetes mellitus (GDM) according to red and processed meat intake stratified by BMI – START cohort

| START (n = 976) | | | | | | | | | | | | | | |
| --- | --- | --- | --- | --- | --- | --- | --- | --- | --- | --- | --- | --- | --- | --- |
| Meat | **BMI <25** | | | | | | | **BMI >25** | | | | | | |
|  | **NC** | | **Low** | | **Medium** | **High** | | **NC** | | **Low** | | **Medium** | **High** | |
|  | **OR** | **(95% CI)** | **OR** | **(95% CI)** |  | **OR** | **(95% CI)** | **OR** | **(95% CI)** | **OR** | **(95% CI)** |  | **OR** | **(95% CI)** |
| Unprocessed Red Meat | - | - | 1.23 | 0.75 - 2.02 | 1 (Ref.) | 1.15 | 0.67 - 1.94 | - | - | 0.60 | 0.31 - 1.19 | 1 (Ref.) | 0.90 | 0.49 - 1.66 |
| Processed Meat | 1.23 | 0.62 - 2.45 | 1.94 | 0.88 - 4.25 | 1 (Ref.) | 1.28 | 0.55 - 2.95 | 0.61 | 0.29 - 1.27 | 1.22 | 0.49 - 3.07 | 1 (Ref.) | 0.69 | 0.29 - 1.64 |
| Total Red and Processed Meat | - | - | 1.22 | 0.49 - 3.07 | 1 (Ref.) | 0.69 | 0.29 - 1.64 | - | - | 0.65 | 0.33 - 1.28 | 1 (Ref.) | 1.02 | 0.55 - 1.89 |

Model adjusted for age, parity, pre-pregnancy BMI, pregnancy weight gain, family history of DM, level of education, total energy, diet quality score, total fiber, saturated fat and glycemic load
