# Supplementary material for: The association of red and processed meat with gestational diabetes mellitus: Results from 2 Canadian birth cohort studies
Source: PLoS One. 2024 May 30;19(5):e0302208. doi: 10.1371/journal.pone.0302208 (PMC11139301; doi:10.1371/journal.pone.0302208)
Supplement: Attachment — Submitted filename: Red Meat and GDM Response Letter_JUL 25 2023.docx [file pone.0302208.s008.docx]

McMaster University

1280 Main Street West, MDCL 3210

Hamilton, Ontario L8S 4K1

July 12, 2023

Emily Chenette, PhD

Editor-in-Chief

PLOS One

**Re: Research article: The association of red meat with gestational diabetes mellitus: results from 2 Canadian birth cohort studies**

Dear Dr. Chenette:

Thank you for taking the time to send our manuscript for review. We value the comments of the reviewers and have made the following changes to the manuscript and responded to the queries posed in the table below.

| **Comments** | **Response** |
| --- | --- |
| This study investigates the association between red meat intake and gestational diabetes mellitus (GDM) risk in two individual cohorts. However, no significant associations were observed in both cohorts. Some improvements can be made before being considered for publication, I list some suggestions and concerns below: | Not applicable |
| ***Introduction*** |  |
| 1. Please include some background information on the meat consumption pattern in Canada and other related regions | Thank you for your comment, we have amended the introduction section of the main paper as follows:  “*This is of keen interest as national cross-sectional survey among select North American countries (US, Canada and Mexico) showed that the median (IQR) grams of unprocessed red meat was higher in Canada (79.0 (36.6–131.6) g), than in the US (72.3 (38.3–124.5) g, p = 0.04) (Frank et al. 2021). Furthermore Canadians (79.0 (36.2–140.1) g) consume higher median intakes of total meat when compared to Mexico (62.5 (31.3–117.4) g, p = 0.04), but not different from median intake in the US (79.4 (40.8–134.7) g).* Page 6, Lines 114 – 120 |
| 2. Line 97-99: “We conducted a prospective … age 18 – 40 years old.” Please revisit this statement as this study did not conduct an actual prospective study | Thank you for this comment, we have clarified the design; the sentence now reads:  “*We explored the association of maternal red and processed meat intake with GDM in two Canadian cohorts of single gestation pregnant women (South Asian and White European) age 18 – 40 years old**.*” Page 6 Lines 121 – 123 |
| ***Methodology*** - The methods section requires more details to ensure clarity |  |
| 1. Please justify the rationale for using the data from 2 cohorts. If I understand correctly from the methodology section, the FAMILY cohort consisted of multiple ethnic groups, including the South East Asian and South Asian. Please justify why only White Europeans were selected from FAMILY, why this study only included the findings from the FAMILY cohort as it consisted of multiple ethnic groups – and further comparison in the associations between red meat intake and GDM among ethnic groups can be made. Besides, there is a time difference between START (2011-2015) and FAMILY cohorts (2004-2009), whereby the eating patterns were somehow different. | Thank you for this comment. Our methods section has been modified and now reads:  “*We opted to use data from these two cohort studies because they represent two different ethnic groups, with different background risks for GDM, and very different dietary patterns. The START cohort includes South Asian Canadians, the largest non-white ethnic group in Canada and FAMILY cohort has data on White Europeans, the largest ethnic group in Canada. It is important to note that though the FAMILY cohort is multi-ethnic. The majority of participants (>70%) are White European. Additionally, both cohort studied assessed diet using a comparable instrument (a validated semi-quantitative food-frequency questionnaire), which improves the comparability of dietary data. Finally, these two cohorts encompassed a wide range of meat intake, which we believed would help us to model the associations more finely.”* Page 7, Lines 142 – 150  Thank you, it’s a very interesting observation with regards to difference in data collection period. We have mentioned this in the discussion as a limitation.  “*Finally, the cohorts represent two time period, roughly 7 years apart. Red (pork, sheep, veal and beef) consumption per capita in Canada between 2004 to 2009 (44.69 kg – 39.93 kg) has decreased when compared to 2011 to 2015 (37.48 kg – 35.54) [48].*” Page 25, Lines 406 – 409 |
| 2. Supplement figure 1: Please give more clear description regarding the flow of participants Consort diagram), some parts of the box cannot be read. | Thank you for this comment. We have listed the starting population for each cohort, and delineated the reasons why participants from each cohort were not included in the present analyses.  We have modified S1 Fig. Sample Population CONSORT Flowchart (see below). |
| 3. Line 196: what type of intake? Total energy intake? Red meat intake? | Thank you we, have modified the sentence to say:  “*START participants had >8-fold lesser mean/ median red and processed meat intake than FAMILY participants; and START had 1.6-fold higher incidence of GDM than FAMILY.*”  Page 10, Lines 229 – 230 |
| 4. I am wondering why the authors use logistic regression for the cohort study since relative risk (RR), and hazard ratio (HR) are more suitable for conducting statistical analysis.  Furthermore, previous studies (lines 85 – 91 & lines 277 - 285) also used RR to perform the analysis. Suggest setting the “medium” group as the reference group. Please confirm and change the statistical analysis. | Thank you, we utilized cross-sectional data from two prospective cohort study, as a result the reporting of odds ratio was more appropriate, we apologize for this confusion.  We have modified the methods section of our paper to that:  “*We used cross-sectional data from two prospective cohort studies…*”. Page 6, Line 126  Additionally, the nature of cross-sectional data for this type of assessment is mentioned in the discussion – limitations.  “*Fourth, in both cohorts, dietary assessment and the OGTT were completed at the same visit (i.e., cross-sectionally), however at the time of FFQ administration, GDM status was not yet known to participants. Additionally, the FFQ inquired about food consumed in the previous 1-year/duration thus, associations are prospectively determined.*” Page 25, Lines 402 – 406  We have modified our analysis to now have medium intake as the reference group, consequently the following sections of the paper has been modified (presented in detail below):   1. Abstract – Results and Conclusion 2. Main manuscript    a. Methods    b. Results    c. Discussion |
| 5. I am confused about how the tertiles (low, medium and high) are determined. Detailed grouping information needs to be provided (e.g., the cut-off for each group). Were those in the low red meat group indeed had low red meat intakes compared to the previous study or standard reference? | Thank you for your comment, we have provided a definition for tertile within the document which reads:  “*Participants in each cohort were placed into three equally-size groups according to cohort-specific tertile cut-offs (refers to any of two points of the ordered distribution of consumers which divides the group into three equal parts) for consumption of red and processed meat (low, medium, and high).”* Page 10, Lines 232 – 235 |
| 6. Line 208 – 216: Further clarification of the multivariate logistic regression is also required. Please explain how the sequence of covariates of the model was made. | Thank you for this comment, we have sought to explain the how the models were made through the following sentences in the document:  “*Covariates entered in the models were chosen based on our reading of previous literature and included both biological and lifestyle / behavioural risk factors for GDM. To build our model, we first used a series of univariate correlation analyses testing variables known or suspected to be associated with either red meat consumption or GDM (e.g. age, prepregnancy BMI, family history of DM, etc.). Any variables associated with either exposure our outcome with a univariate correlation p<0.20 were considered for inclusion. Then, we present models sequentially adjusted for more variables, entered as sets representing an underlying concept (e.g., non-modifiable risk factors; body weight; lifestyle factors; and dietary variables).”* Pages 10 - 11, Lines 246 – 254 |
|  |  |
| ***Results*** |  |
| 1. Tables: Please refer to the journal guideline on the presentation of tables. | Thank you for this comment, we have included all tables within the main document directly after the paragraph in which it is first cited.  Pages 12-14 – Table 1  Pages 15-17 – Table 2  Page 19 – Table 3 |
| 2. Table 1: Please include n (%) for each category. | Thank you for highlighting this, Table 1 has been modified to include n (%) for each category. Page 12 |
| 3. Table 2: Diet index (last row). Diet quality index (please use a standardized term throughout the entire manuscript)? If not, please include a description in the Methodology section | Thank you for this observation we speak about both diet quality and diet index (which is a researcher derived index score from a previous study) we have now modified the methods section of the main document to state:  “*To describe overall diet quality, we used a previously developed diet quality score (DQS), calculated as the sum of daily servings of “healthy” foods (fermented dairy, fish and seafood, leafy green vegetables, cruciferous vegetables, legumes, fruits, nuts, and whole grains) less the sum of daily servings of “unhealthy foods” (processed meats, refined grains, French fries, snacks, sweets, and sweet drinks)*.” Pages 8 - 9, Lines 192 – 196 |
| 4. Suggest combining paragraphs 1 and 2 of the results and simplifying it | Thank you for this suggestion, we have combined paragraphs 1 and 2, and also made modified overall.  *“**Tables 1 and 2 show the participants’ demographic characteristics and dietary factors by intake of total red meat, separately for START and FAMILY (see Supplemental 3 for unprocessed red meat and processed meat). The median intake of unprocessed red meat was 1.34 g/d (0.2-11.0 g/d); of processed meat 0.0 g/d (0.0-0.2 g/d); and of total red and processed meat 1.5 g/d (0.2-11.3 g/d) in START (n=976). The median intake of unprocessed red meat was 44.1 g/d (27.5-65.9 g/d); of processed meat was 11.5 g/d (6.2-19.7 g/d); and of total red and processed meat was 58.2 g/d (37.1-85.7 g/d) in FAMILY (n=581). 24.7% (n = 241) of START participants and 15.7% (n = 91) of FAMILY participants were diagnosed with GDM (Supplemental 3). FAMILY participants when compared to START participants were younger and likely to smoke (currently or previously). Within the FAMILY cohort, high total red and processed meat consumers were less likely to be married or in a common-law relationship, and had lower levels of education, employment, and physical activity. START participants who had high total red and processed meat intake were more likely to be unemployed or retired, and have an annual household income >$50,000 compared to their low total red and processed meat consumer counterparts. In both cohorts, high total red and processed meat consumers had a higher pre-pregnancy BMI, and a higher SDI. Higher total red and processed meat consumers were more likely to have a family history of T2D in START.”* Page11, Lines 263 - 279 |
| 5. Line 240 – 247: Please revisit this paragraph, as it is confusing. | We have modified the paragraph and it now reads:  “*Red and processed meat consumption was associated with other nutrients and food groups that may influence cardiometabolic health. For example, high total red and processed meat consumers in START consumed higher amounts of total protein, total fat, cholesterol, and less total fibre (Table 2). High total red and processed meat consumers in FAMILY consumed higher total energy, total protein, total fat, saturated fat, cholesterol, and less total carbohydrates, total fibre, and sugar compared to low consumers. In START, higher consumers of total red and processed meat ate more poultry, fish, seafood, and eggs; and less dairy and legumes. In FAMILY, higher consumers of total red meat ate more poultry, fish, seafood, dairy, eggs, and legumes; but lower amounts of nuts*.” Page 18, Lines 239 – 247 |
| 6. Line 248-259: Please revisit this paragraph, as it did not reflect all the findings in Table 2 | Thank for your comment section of the main document has been modified as follows:  “*In START, neither lower nor higher intakes of unprocessed red meat, processed meat, or total red and processed meat were associated with increased risk of GDM, when compared with medium intakes (Table 3), with no evidence of a trend across categories. In FAMILY, only processed meat was associated with development of GDM (p-trend = 0.04), but this was attenuated with adjustment for additional dietary factors such as the diet quality score, total fiber, saturated fat, and glycemic load. The test for multicollinearity did not detect any significant correlation (all variance inflation factor values were <2 and tolerance >0.7) between covariates in the fully adjusted model for both START and FAMILY cohorts.*” Page 18, Lines 248 - 255 |
| ***Discussion*** |  |
| 1. Line 270-272: This finding was not shown in the result section. | Thank you for your comment. The sentence has been removed from the manuscript. |
| 2. Line 295-299: Could the authors comment on why white Europeans had higher intakes of refined grains, and lower intakes of fiber (based on 299 a 2000 kcal diet) than South Asians, but had a lower prevalence of GDM? | Thank you that’s an interesting question. We have included a sentence which makes reference to the potential impact genetics.  *“This indicates that they are other factors (such as genetics) associated with GDM in the South Asian cohort. Lamri [32] studied pregnant South Asian women from two birth cohort studies, and reported that increased polygenic risk score was associated with 45% increased risk of GDM, with adjustment for* *low diet quality and parental history of T2D.”* Page 22, Lines 299 – 303 |
| 3. Line 324: what is the cut-off for moderation spoused by most dietetic and health organizations, and are any references for this? | Thank you for this query ww have modified the manuscript.  *“Though several countries recommend the moderate consumption of meat there is much ambiguity with regards to the number of servings along with portion size which constitutes ‘moderation’. Food-based dietary guidelines within Europe do offer some guidance but the recommendations vary from having 1 serving of red meat per week with a serving size of 120 – 150g (Greece) to reducing consumption to 70g/d for individuals who consume more than 90g/d red and/ or processed meat (United Kingdom) [43]. The Dietary Guideline for the Americas [44] recommends for its population consuming 26 ounces equivalent /wk for meats, poultry, eggs based on a 2,000 calorie diet. However, it is important to note that there is a running theme with the promotion of more lean cuts of red meat or processed meat. In India, there is a general recommendation of moderate consumption of meat [45]. The Canadian dietary guidelines make no reference to meat consumption, however, it recommends that the general population (2 years and older) should “choose protein foods that come from plants more often, and try to eat food with healthy fats instead of saturated fat, as well as to limit highly processed foods in small amounts.” [46].”* Page 23, Lines 330 - 343 |

Throughout the manuscript we have additional amendments:

1. Abstract:
   a. Objective - Red and processed meat is considered risk factors of gestational diabetes mellitus (GDM), but the evidence is inconclusive. Page 3, Lines 53 - 54
   b. Results - In START, the multivariable-adjusted odds ratio (OR) showed neither lower nor higher intakes of unprocessed red meat (p-trend = 0.68), processed meat (p-trend = 0.90), or total red and processed meat (p-trend = 0.44), were associated with increased risk of GDM, when compared with medium intake. Similar results were observed in FAMILY except for processed meat intake [OR = 0.94 (95% CI 0.47 – 1.91), for medium versus low and OR = 1.51 (95% CI 0.77 – 2.29) for medium versus high; p-trend = 0.18] after adjusting for additional dietary factors such as the diet quality score, total fiber, saturated fat and glycemic load. Page 3, Lines 64 - 71
   c. Conclusion - Medium compared with lower or high red and processed meat intake is not associated with GDM in White Europeans and South Asians living in Canada. Page 3, Lines 72 – 73
2. Take away messages:
   a. South Asian women consumed less red and processed meat than White Europeans but are more likely to develop gestational diabetes. Page 4, Lines 78 - 79
   b. We found no statistically significant association between total red and processed meat consumption and odds of gestational diabetes in two birth cohort studies in Southern Ontario. Page 4, Lines 80 - 81
3. Main Manuscript - Results:
   a. In START, neither lower nor higher intakes of unprocessed red meat, processed meat, or total red and processed meat were associated with increased risk of GDM, when compared with medium intakes (Table 3), with no evidence of a trend across categories. In FAMILY, only processed meat was associated with development of GDM (p-trend = 0.04), but this was attenuated with adjustment for additional dietary factors such as the diet quality score, total fiber, saturated fat, and glycemic load. The test for multicollinearity did not detect any significant correlation (all variance inflation factor values were <2 and tolerance >0.7) between covariates in the fully adjusted model for both START and FAMILY cohorts. Page 18, Line 248 – 255
4. Main Manuscript - Discussion:
   a. In this analysis of two prospective birth cohort studies, we observed that medium compared with non-consumption, low or high intake of unprocessed red meat, processed meat, or total red meat was not associated with increased risk of GDM. Page 21, Line 271 – 273
   b. As with most dietary recommendations for moderate red meat intake we used medium intake as our reference group which is more reflective contextually. Page 24, Line 378 – 380
5. Main Manuscript - Conclusion:
   a. The results indicate that medium compared with low or high red and processed meat intakes were not associated with GDM among White Europeans or South Asians (despite the high percentage of non-consumers and low consumption) living in Canada. Page 25, Line 413 – 416
6. References:
   a. Frank, Sarah M., Jaacks, Lindsay M., Batis, Carolina, Vanderlee, Lana, Taillie, Lindsey S. Patterns of Red and Processed Meat Consumption across North America: A Nationally Representative Cross-Sectional Comparison of Dietary Recalls from Canada, Mexico, and the United States. Int J Environ Res Public Health. 2021;18. doi:10.3390/ijerph18010357 - # 16
   b. Lamri A, Limbachia J, Schulze KM, Desai D, Kelly B, de Souza RJ, et al. The genetic risk of gestational diabetes in South Asian women. Janus ED, Azziz R, Janus ED, editors. eLife. 2022;11: e81498. doi:10.7554/eLife.81498 - #31
   c. Health Promotion and Disease Prevention Knowledge Gateway. Food-based dietary guidelines in Europe - Table 8. European Commission; 2021. Available: https://knowledge4policy.ec.europa.eu/health-promotion-knowledge-gateway/food-based-dietary-guidelines-europe-table-8_en - #43
   d. U.S. Department of Agriculture and U.S. Department of Health and Human Services. Dietary Guidelines for Americans, 2020-2025. 9th edition. 2020 Dec. Available: https://www.dietaryguidelines.gov/sites/default/files/2021-03/Dietary_Guidelines_for_Americans-2020-2025.pdf - #44
   e. ICMR-National Institute of Nutrition. Nutrition, Lifestyle and Immunity. Available: https://www.nin.res.in/downloads/Nutrition_Lifestyle_and_Immunity.pdf - #45
   f. Health Canada. Canada’s Dietary Guidelines for Health Professional and Policy Makers. 2019. Available: https://food-guide.canada.ca/sites/default/files/artifact-pdf/CDG-EN-2018.pdf - #46
   g. Organisation for Economic Co-operation and Development [OECD]. Meat consumption (indicator). 2023. doi:10.1787/fa290fd0-en - #48
7. Conflict of Interests:
   a. HCG holds the McMaster-Sanofi Population Health Institute Chair in Diabetes Research and Care. He reports research grants from Eli Lilly, AstraZeneca, Novo Nordisk, and Sanofi; honoraria for speaking from AstraZeneca, Boehringer Ingelheim, Eli Lilly, Novo Nordisk, DKSH, Zuellig, Roche, Sanofi, Jiangsu Hanson, and Carbon Brand; and consulting fees from Abbott, Eli Lilly, Novo Nordisk, Sanofi, Kowa, Pfizer, Hanmi and Viatris. Page 26, Line 439 – 443
8. Data Availability:
   a. All relevant data are within the paper and its Supporting Information files. Raw data cannot be shared publicly because of lack of participant consent, but are available upon request to Natalie Williams, McMaster University at: Email: [natcampb@mcmaster.ca](mailto:natcampb@mcmaster.ca) . Page 27, Lines 462 - 465
9. S2 Table 1. Listing of Red Meat and Processed Meat Classification by Cohorts – added

| **Food Groups** | **FAMILY Food Items** | **START Food Items** |
| --- | --- | --- |
| Red Meat | Ground beef  Roast beef  Steak  Pot roast  Pork chop  Baked ham  Veal  Lamb  Lunch ham /corned beef | Roast beef /steak  Pork chop /mince /keema /kabob /dry kofta  Ground beef /mince /hamburger /keema kabob /dry kofta  Goat /lamb mince /roast /steak /chop /keema /kabob /dry kofta /raan  Beef curry /kofta  Pork curry /kofta  Goat /lamb curry |
| Likely Red Meat, Processed  (80% RM unless otherwise specified) | Hot dog  Sausage  Other lunch meat  Bacon  Pickled meat /fish (50%)*  Salted /dried meat /fish (50%)* | Hot dog /sausage  Lunch meat |

*We assumed that 50% of these two categories were servings of red meat and were counted as such.

10. S1 Fig. Sample Population CONSORT Flowchart

**FAMILY**

N = 857

N

N= 857

**Population for Analysis**

n = 581

**Excluded = 276**

Mothers of multiples, n = 41

Non-white ethnicity, n = 133

History of Diabetes, n = 37

Age >40, n =29; Age<18, n =5

Caloric Intake missing or outside [500,4500], n = 92

**START**

N = 1,012

N

N= 857

**Population for Analysis**

n = 976

**Excluded = 36**

Mothers of multiples, n = 0

Early withdrawal, n = 2

History of Diabetes, n = 4

Age >40, n = 4; Age<18, n = 0

Caloric Intake missing or outside [500,4500], n = 27

Note:

OGTT = oral glucose tolerance test

Yours truly,

Russell de Souza

[desouzrj@mcmaster.ca](mailto:desouzrj@mcmaster.ca)
