# Supplementary material for: The association of red and processed meat with gestational diabetes mellitus: Results from 2 Canadian birth cohort studies
Source: PLoS One. 2024 May 30;19(5):e0302208. doi: 10.1371/journal.pone.0302208 (PMC11139301; doi:10.1371/journal.pone.0302208)
Supplement: Attachment — Submitted filename: red-meat-GDM-Review-R.docx [file pone.0302208.s009.docx]

**Review**

In the introduction, I am not clear why they chose these 2 Canadian cohorts from different ethnic groups. I would suggest that the authors should build up the case why they chose the 2 cohorts with references (several previous studies are there) , highlighting most number of studies done in US, association of consumption of PM, UM with GDM is not very clear in Asian population with reference etc. The authors have rightly mentioned that the evidence on the association between dietary animal protein sources and GDM is inconsistent, but they haven’t mentioned whether there were any methodological flaws/deficiencies in the previous studies and that they have addressed in this analysis to add to the evidence

In the methodology

*Line 127-128: We used cross-sectional data from two prospective cohort studies: the SouTh Asian birth cohorT (START) .. FAMILY*

Have they used cross sectional data? What I understand from the paper is that they have used prospective cohort data, measured diet data of 12 months before pregnancy and followed up incident GDM and co-variates measured in the 2^nd^ trimester. Analysed retrospectively.

line 129-131, I suggest that the overall objective of the START cohort study is mentioned like they have discussed about FAMILY study in lines 136-137

Line 148-149*,* I suggest that authors mention which validated semi-quantitative food-frequency questionnaire was used in these 2 cohort studies,(was SHARE FFQ in both studies?) how many items, any difference in method of analyses

*Line 150-151* Though the authors argued that the *‘two cohorts encompassed a wide range of meat intake, which we believed would help model the associations more finely’,* they finally decided to analyse separately the data from 2 cohorts(*decided to examine the data based on cohort-specific intake of red and processed meat)* as they found START cohort with lower meat consumption reported higher prevalence of GDM. Didn’t the sample size have sufficient power to compare the association between different major ethnic groups and tease out the major confounders, effect modifiers to make this paper more valuable?

Lines 157 to 160 There isn’t sufficient explanation to say why the authors decided to analyse only data of white European from FAMILY cohort study especially there were south Asians in that cohort who were a major ethnic group the analysis is focusing on.

*Line- 182-183 Recipes were generated for red and processed meat composite food items that were not available in the database, and a nutrient value/ profile was developed.*

Lines 233-236: Why did the authors decide to place 3 equally-size groups? They were basically comparing within the group. To be biologically plausible, wasn’t it good to place them in respective tertiles by defining the high, medium and low intake instead of cohort specific tertile cut-offs.

Line 237-240: As more than 50% of the participants in START were non-consumers of processed meat, using cohort specific tertile cut-offs for the remaining 239 participants is not correct. Should have used a definition for high , medium and low intake to prove a biologically plausible association

In summary there are several methodological issues in this study. My recommendation is to reanalyse and revise the manuscript.
