# Supplementary material for: The association of red and processed meat with gestational diabetes mellitus: Results from 2 Canadian birth cohort studies
Source: PLoS One. 2024 May 30;19(5):e0302208. doi: 10.1371/journal.pone.0302208 (PMC11139301; doi:10.1371/journal.pone.0302208)
Supplement: Attachment — Submitted filename: Red-meat-GDM Response Letter_Oct 27.docx [file pone.0302208.s010.docx]

McMaster University

1280 Main Street West, MDCL 3210

Hamilton, Ontario L8S 4K1

October 24, 2023

Emily Chenette, PhD

Editor-in-Chief

PLOS One

**Re: Research article: The association of red meat with gestational diabetes mellitus: results from 2 Canadian birth cohort studies**

Dear Dr. Chenette:

Thank you for taking the time to send our manuscript for review. We value the comments of the reviewers and have made the following changes to the manuscript and responded to the queries posed in the table below.

| **Comments** | **Response** |
| --- | --- |
|  |  |
| ***Introduction*** |  |
| In the introduction, I am not clear why they chose these 2 Canadian cohorts from different ethnic groups. I would suggest that the authors should build up the case why they chose the 2 cohorts with references (several previous studies are there), highlighting most number of studies done in US, association of consumption of PM, UM with GDM is not very clear in Asian population with reference etc. The authors have rightly mentioned that the evidence on the association between dietary animal protein sources and GDM is inconsistent, but they haven’t mentioned whether there were any methodological flaws/deficiencies in the previous studies and that they have addressed in this analysis to add to the evidence. | Thank you for your comment, we have amended the introduction and discussion sections of the main paper as follows:  Introduction  *Ethnicity and diet are risk factors for GDM [10–17]. Women of African, Arab, Asian, Hispanic, Indigenous, Native American, and South Asian ethnicity are at increased risk of developing GDM. Farrar et al. [18], in the UK-based “Born-in-Bradford” birth cohort study among Europeans and South Asian women, found that the prevalence of GDM among South Asians (range 4.1 – 17.4%) was approximately twice that of White British counterparts (range: 1.2 – 8.7%), irrespective of the diagnostic cut-off threshold used. Anand et al. [19] reported a 36.3% prevalence of GDM among 1006 expectant South Asian mothers in Canada using the ethnic-specific Born-in-Bradford (BiB) criteria. However, there is uncertainty about the exact role/mechanism of some risk factors such as ethnicity in the development of GDM.* Page 5, lines 101 – 110  Discussion  *Furthermore, previous studies of red and processed meat and GDM risk have not included large numbers of high-risk ethnic groups. Our South Asian birth cohort is one the largest cohorts of pregnant women in North America, and ideally suited to assess the robustness of associations observed in White Europeans.* Page 25, lines 382 – 385 |
| ***Methodology*** |  |
| *Line 127-128: We used cross-sectional data from two prospective cohort studies: the SouTh Asian birth cohorT (START) .. FAMILY*  Have they used cross sectional data? What I understand from the paper is that they have used prospective cohort data, measured diet data of 12 months before pregnancy and followed up incident GDM and co-variates measured in the 2^nd^ trimester. Analysed retrospectively. | Thank you for this comment. Though both birth cohorts are prospective (FAMILY and START) participants completed the food-frequency questionnaire at the same visit at which they took the OGTT (i.e., at a cross-section in time), thus the analysis is not a “clean” prospective design. We agree, participants are recalling diet prior to the OGTT results, however, to be true to the data collection approach, we have chosen to present the more conservative analysis that reflects this. We have stated this in the methods section:  “*We used cross-sectional data from two prospective cohort studies…*”. Page 6, line 136  Additionally, the nature of cross-sectional data for this type of assessment in our study is mentioned in the discussion – limitations section.  “*Fourth, in both cohorts, dietary assessment and the OGTT were completed at the same visit (i.e., cross-sectionally), however at the time of FFQ administration, GDM status was not yet known to participants. Additionally, the FFQ inquired about food consumed in the previous 1-year/duration thus, associations are prospectively determined.*” Page 26, lines 406 – 410 |
| line 129-131, I suggest that the overall objective of the START cohort study is mentioned like they have discussed about FAMILY study in lines 136-137 | Thank you for this comment. We have included the following sentence:  *The study seeks to understand the role of fetal programming in the development of adiposity in South Asians in general - a high risk group for T2DM and CVD.* Pages 6 - 7, lines 140 - 141 |
| Line 148-149*,* I suggest that authors mention which validated semi-quantitative food-frequency questionnaire was used in these 2 cohort studies, (was SHARE FFQ in both studies?) how many items, any difference in method of analyses | Thank you, we have modified the methods section of the paper to say:  “*The Study of Health and Risk in Ethnic Groups Food Frequency Questionnaires (SHARE-FFQs) was administered at the initial visit in both cohort studies (second to third trimester). The validated semi-quantitative 163-item SHARE-FFQ was developed for South Asians - START (163-item) and White Europeans - FAMILY (157-item) living in Canada. [29]. Briefly, for FFQs participants are asked to indicate their frequency of consumption of each item over the past 12-months (per day, per week, per month, per year, or never), with three reference serving sizes provided. A picture or common measure was shown for a medium, or typical serving, and a small serving was half the size of a medium, and a large was 1.5 to 2-times the size of a medium [29]. Reproducibility and validity of the SHARE-FFQ can be found in other publications [29–31].*” Page 8, lines 183 – 193  Additionally, we have also moved the following sentence to the end of the paragraph:  “*Nutrient intake obtained from the SHARE-FFQs was calculated using the Food Processor nutrient analysis software (version 6.11, 1996, ESHA, Salem, OR), derived from the 1991 Canadian Nutrient File (CFN) and the US Department of Agriculture nutrient food composition databases. The updated 1997 CFN was used to ascertain meat and poultry nutrient values. Recipes were generated for red and processed meat composite food items that were not available in the database, and a nutrient value/ profile was developed. Because serving size varies across food items (e.g., a serving of a hotdog or lunch meat is smaller than a serving of steak or meatloaf) and some items (e.g., ground beef as hamburger, meatloaf, in casseroles) are not entirely meat, the estimated average grams of meat per serving for each food item was calculated and standardized by a registered dietitian familiar with South Asian and European food items. Grams of meat for each sub-item were totalled to get an estimate for each composite item (e.g., baked ham is 100% meat; ground beef as hamburger, meatloaf, in casseroles is 84% meat).*” Page 8, lines 194 - 205 |
| *Line 150-151* Though the authors argued that the *‘two cohorts encompassed a wide range of meat intake, which we believed would help model the associations more finely’,* they finally decided to analyse separately the data from 2 cohorts(*decided to examine the data based on cohort-specific intake of red and processed meat)* as they found START cohort with lower meat consumption reported higher prevalence of GDM. Didn’t the sample size have sufficient power to compare the association between different major ethnic groups and tease out the major confounders, effect modifiers to make this paper more valuable? | Thank you for your comment we have included the following sentences in the methods section:  “*Additionally, both cohort studies assessed diet using a comparable instrument (SHARE FFQs - a validated semi-quantitative food-frequency questionnaires), which improved the comparability of dietary data. To account for the intractable association/ confounding with red and processed meat intake and ethnicity (i.e., cohort) we created cohort-specific groups.*” Page 7, lines 162 - 165  Regarding sample size and power calculations, we did not intend to compare the direct association of red and processed meat intake in the original birth cohort studies. Indeed, given our fixed sample size and number of cases we do not believe that a larger sample size would have been more robust in our analysis. Our results are comparable to that of the Nurse’s Health Study 2 by Zhang et al. (2006)^[1]^ (a larger cohort study with 758 GDM cases of 13,110 participants).  In our power calculation for this study, we considered previous studies on the association between red and processed meat and GDM specifically we were guided by data in the Zhang et al. (2006). In this study higher compared with lower intake of red and processed meat were assessed for their association with GDM. These associations were assessed on the background of western and prudent dietary pattern. The western dietary pattern is very similar to that of the participants in FAMILY and the prudent is similar to START. The contrast between high and low for the western group was 88.4 (total red + processed meat), while the contrast between our (FAMILY) high and low was 66.4g, similarly the contract their high and low meat in the prudent group was 22.95g and ours (START) was 20.42g (see table 1 below).  In the Zhang et al. (2006) paper the magnitude of the RR was 1.63 (western) and 1.39 (prudent) in ours our high vs low was OR was 1.63 (FAMILY) and 1.14 (START).  However, at the request of a previous reviewer we change the reference group from low to medium for ease of interpretation.  In our study with 91/581 cases – FAMILY and 241/976 cases START, we have 80% power to detect an OR of at least 1.74 in FAMILY and, in START an odds ratio as small as 1.41. With 758/13,110 (5.8%) cases, Zhang et al. report a 95% confidence interval of the observed relative risk, comparing a western and prudent diet, to range from 1.09 to 2.21. We are sufficiently powered to detect associations consistent within this range.  We do not believe that a statistical test of interaction would be of value because we are not testing the hypothesis of between-cohorts interaction. We are assuming that the cohorts will be different because of their sample characteristics, and the distribution of the outcomes and exposure. This is why we decided to treat the cohorts separately, and not test for interaction formally.  However, the issue of sample size and power calculation is address in the discussion – limitation section of the paper:  “*Third, our sample size was small, which reduced our power in multivariable analyses. Nevertheless, our point estimates in FAMILY are aligned with larger studies which have reported positive associations between red and processed meats and GDM [21,22,24]. We also had a relatively high event proportion or GDM (16% - FAMILY and 25% - START) compared to other studies which had approximately 5% [21,22,24,25]*.”  Page 26, lines 402 - 406 |
| Lines 157 to 160 There isn’t sufficient explanation to say why the authors decided to analyse only data of white European from FAMILY cohort study especially there were south Asians in that cohort who were a major ethnic group the analysis is focusing on. | Thank you for this comment, we have included the following:  “*The START cohort included South Asian Canadians, the largest non-white ethnic group in Canada while the FAMILY cohort included White Europeans, the largest ethnic group in Canada., although the FAMILY cohort was multi-ethnic [White European, East or South East Asian, Aboriginal, South Asian, and African or other origins] [28], the majority of participants (>80%) are White European, as a result we opted to exclude other ethnic groups (which includes approximately 2% South Asians) for our analysis*” Page 7, lines 155 – 161.  Furthermore, only 2.1% (n = 17) in FAMILY was South Asian and 0% (n = 0) of White European in START. Within the FAMILY cohort we had only 5 cases of GDM of the 17 South Asian participants (before applying our inclusion and exclusion criteria). Given this we opted not to included South Asians of the FAMILY cohort in our South Asian population for this analysis.  Sensitivity analysis of the difference in odds ratio if we were to include South Asian (15 once the study inclusion and exclusion criteria is applied) from the FAMILY in the START population revealed the results would have been similar (see tables 2a and 2b below). |
| *Line- 182-183 Recipes were generated for red and processed meat composite food items that were not available in the database, and a nutrient value/ profile was developed.* | N/A |
| Lines 233-236: Why did the authors decide to place 3 equally-size groups? They were basically comparing within the group. To be biologically plausible, wasn’t it good to place them in respective tertiles by defining the high, medium and low intake instead of cohort specific tertile cut-offs. | Thank you for this question. Food intake is not normally distributed (positively skewed; all values >=0) and by placing people into categories of red and processed meat intake we minimize the impact of outliers and ensure equal group size at each exposure level, and those optimize our power and stability of risk estimates within the groups.  Nutritional epidemiology has a long history with this approach and therefore our results will be more comparable to other studies.  The following has been added to the paper:  “*By creating cohort-specific tertile cut-offs and acknowledging the large number of processed meat non-consumers in the START cohort we minimize the impact of outliers and ensure equal group size at each exposure level, and those optimize our power and stability of risk estimates within the groups.*”  Page 11, lines 269 - 273 |
| Line 237-240: As more than 50% of the participants in START were non-consumers of processed meat, using cohort specific tertile cut-offs for the remaining 239 participants is not correct. Should have used a definition for high, medium and low intake to prove a biologically plausible association | Thank you for your comment within the manuscript it states:  Participants in each cohort were placed into three equally-size groups according to cohort-specific tertile cut-offs (refers to any of two points of the ordered distribution of consumers which divides the group into three equal parts) for consumption of unprocessed red meat and total red and processed meat (low, medium, and high). Processed meat was handled differently. In START, most participants (56.9%, n=565/976) reported consuming 0 g/d of processed meat. Therefore, we created a non-consumer group and placed the remaining participants into groups based on tertile cut-off points (low, medium, and high consumers). Pages 10, lines 263 – 269  The following has been added to the paper:  “*By creating cohort-specific tertile cut-offs and acknowledging the large number of processed meat non-consumers in the START cohort we minimize the impact of outliers and ensure equal group size at each exposure level, and those optimize our power and stability of risk estimates within the groups.*” Page 11, lines 269 - 273 |
| In summary there are several methodological issues in this study. My recommendation is to reanalyse and revise the manuscript. | N/A |
| Editorial |  |
| Addition of Reference | The following references have been added to the manuscript:  10. Berkowitz GS, Lapinski RH, Wein R, Lee D. Race/ethnicity and other risk factors for gestational diabetes. Am J Epidemiol. 1992;135: 965–973.  11. Feig DS, Berger H, Donovan L, Godbout A, Kader T, Keely E, et al. Diabetes and Pregnancy. Can J Diabetes. 2018;42: S255–S282. doi:10.1016/j.jcjd.2017.10.038  12. Giannakou K, Evangelou E, Yiallouros P, Christophi CA, Middleton N, Papatheodorou E, et al. Risk factors for gestational diabetes: An umbrella review of meta-analyses of observational studies. PLOS ONE. 2019;14: e0215372. doi:10.1371/journal.pone.0215372  13. Mijatovic-Vukas J, Capling L, Cheng S, Stamatakis E, Louie J, Cheung NW, et al. Associations of Diet and Physical Activity with Risk for Gestational Diabetes Mellitus: A Systematic Review and Meta-Analysis. Nutrients. 2018;10. doi:10.3390/nu10060698  14. Schoenaker DAJM, Mishra GD, Callaway LK, Soedamah-Muthu SS. The Role of Energy, Nutrients, Foods, and Dietary Patterns in the Development of Gestational Diabetes Mellitus: A Systematic Review of Observational Studies. Diabetes Care. 2016;39: 16. doi:10.2337/dc15-0540  15. Teh WT, Teede HJ, Paul E, Harrison CL, Wallace EM, Allan C. Risk factors for gestational diabetes mellitus: Implications for the application of screening guidelines. Aust N Z J Obstet Gynaecol. 2011;51: 26–30. doi:10.1111/j.1479-828X.2011.01292.x  16. Yuen L, Wong VW. Gestational diabetes mellitus: challenges for different ethnic groups. World J Diabetes. 2015;6: 1024–1032. doi:10.4239/wjd.v6.i8.1024 Yuen L, Wong VW. Gestational diabetes mellitus: Challenges for different ethnic groups. World J Diabetes 2015; 6(8): 1024-1032 [PMID: 26240699 DOI: 10.4239/wjd.v6.i8.1024]  17. Zhang C, Ning Y. Effect of dietary and lifestyle factors on the risk of gestational diabetes: review of epidemiologic evidence. Am J Clin Nutr. 2011;94: 1975S-1979S. doi:10.3945/ajcn.110.00103 |

Yours truly,

Russell de Souza

[desouzrj@mcmaster.ca](mailto:desouzrj@mcmaster.ca)

Table 1: Nurse’s Health 2 versus FAMILY and START Study Characteristics

|  | **Western Diet*** | | | **Prudent Diet*** | | | **FAMILY** | | | **START** | | |
| --- | --- | --- | --- | --- | --- | --- | --- | --- | --- | --- | --- | --- |
|  | **low** | **high** | **difference** | **low** | **high** | **difference** | **low** | **high** | **difference** | **low** | **high** | **difference** |
| **Red + Processed Meat (g)** | 26.35 | 114.75 | 88.4 | 75.65 | 52.7 | 22.95 | 30.3 | 96.7 | 66.4 | 0.06 | 20.48 | 20.42 |
| **GDM** | (17.3%) | | | (17.3%) | | | (15.7%) | | | (24.7%) | | |
| **RR/ OR** | high vs low = RR = 1.63 (1.20 - 2.21, p=0.0011) | | | low vs high = RR = 1.39 (1.09 - 1.72, p=0.018) | | | high vs medium = OR = 1.19 (0.61 - 2.31, p = 0.30) | | | high vs medium = OR = 1.12 (0.77 - 1.65, p = 0.44) | | |
| **Model** | Adjusted for age, parity, BMI, race/ethnicity, cigarette smoking status, family history of diabetes in a first-degree relative, alcohol intake, physical activity and total energy | | | | | | Adjusted for age, parity, pre-pregnancy BMI, pregnancy weight gain, smoking (FAMILY only), family history of DM, level of education, total energy, diet quality score, total fiber, saturated fat and glycemic load | | | | | |

*85 g per serving

Table 2a: Association between low and high red and processed meat intake relative to medium intake and gestational diabetes mellitus (GDM) by Cohort

| Variable | FAMILY | | | | | | START | | | | | | | |
| --- | --- | --- | --- | --- | --- | --- | --- | --- | --- | --- | --- | --- | --- | --- |
|  | **Low** | | **Medium** | **High** | | **p-trend** | **Non-consumer** | | **Low** | | **Medium** | **High** | | **p-trend** |
|  | **OR** | **95% CI** |  | **OR** | **95% CI** |  | **OR** | **95% CI** | **OR** | **95% CI** |  | **OR** | **95% CI** |  |
| Total Red and Processed Meat median (g/d) | **30.3** | | **58.2** | **96.7** | |  | **-** | - | **0.06** | | **1.5** | **20.5** | |  |
| GDM cases/ pregnancies | 24/193 | | 32/194 | 35/194 | |  | - | - | 75/325 | | 80/326 | 86/325 | |  |
| Fully adjusted model | 0.69 | 0.35 - 1.36 | 1 (Ref.) | 1.19 | 0.61 - 2.31 | 0.30 | - | - | 1.05 | 0.72 - 1.55 | 1 (Ref.) | 1.12 | 0.77 - 1.65 | 0.44 |

Model: Adjusted for age, parity, pre-pregnancy BMI, pregnancy weight gain, smoking (FAMILY only), family history of DM, level of education, total energy, diet quality score, total fiber, saturated fat and glycemic load

Table 2b: Association between low and high red and processed meat intake relative to medium intake and gestational diabetes mellitus (GDM) by Ethnicity (White European versus South Asian)

| Variable | White European | | | | | | South Asian | | | | | | | |
| --- | --- | --- | --- | --- | --- | --- | --- | --- | --- | --- | --- | --- | --- | --- |
|  | **Low** | | **Medium** | **High** | | **p-trend** | **Non-consumer** | | **Low** | | **Medium** | **High** | | **p-trend** |
|  | **OR** | **95% CI** |  | **OR** | **95% CI** |  | **OR** | **95% CI** | **OR** | **95% CI** |  | **OR** | **95% CI** |  |
| Total Red and Processed Meat median (g/d) | **30.3** | | **58.2** | **96.7** | |  | **-** | - | **0.06** | | **1.5** | **20.5** | |  |
| GDM cases/ pregnancies | 24/193 | | 32/194 | 35/194 | |  | - | - | 77/334 | | 81/329 | 87/328 | |  |
| Fully adjusted model | 0.69 | 0.35 - 1.36 | 1 (Ref.) | 1.19 | 0.61 - 2.31 | 0.30 | - | - | 1.03 | 0.70 – 1.52 | 1 (Ref.) | 1.12 | 0.78 – 1.74 | 0.63 |

Model 4: Adjusted for age, parity, pre-pregnancy BMI, pregnancy weight gain, smoking (White European only), family history of DM, level of education, total energy, diet quality score, total fiber, saturated fat and glycemic load

Note: 15 participants from the FAMILY cohort was added to the South Asian group from START

1. Zhang C, Schulze MB, Solomon CG, Hu FB. A prospective study of dietary patterns, meat intake and the risk of gestational diabetes mellitus. Diabetologia. 2006;49: 2604–2613. doi:10.1007/s00125-006-0422-1
