# Supplementary material for: The association of red and processed meat with gestational diabetes mellitus: Results from 2 Canadian birth cohort studies
Source: PLoS One. 2024 May 30;19(5):e0302208. doi: 10.1371/journal.pone.0302208 (PMC11139301; doi:10.1371/journal.pone.0302208)
Supplement: Attachment — Submitted filename: red-meat-GDM-Review-R-2.docx [file pone.0302208.s011.docx]

**Review**

Thank you for asking me to review this interesting paper after revision.

The authors have revised the introduction with better clarity of the cohorts chosen, the composition of the cohorts and reason for choosing the respective cohorts. The authors have clarified all the queries raised in the methods and statistical analysis section. They have described the best possible methods used for dietary assessment and analysis of data using traditional methods in nutrition epidemiology with its own limitations. Though this study has negative results, it will make significant contribution to tease out the inconsistent association found between red meat, processed meat, unprocessed meat and GDM.

It can be considered for publication.
