# Supplementary material for: The association of red and processed meat with gestational diabetes mellitus: Results from 2 Canadian birth cohort studies
Source: PLoS One. 2024 May 30;19(5):e0302208. doi: 10.1371/journal.pone.0302208 (PMC11139301; doi:10.1371/journal.pone.0302208)
Supplement: Attachment — Submitted filename: Reviewer comments.docx [file pone.0302208.s007.docx]

This study investigates the association between red meat intake and gestational diabetes mellitus (GDM) risk in two individual cohorts. However, no significant associations were observed in both cohorts. Some improvements can be made before being considered for publication, I list some suggestions and concerns below:

Introduction

1. Please include some background information on the meat consumption pattern in Canada and other related regions
2. Line 97-99: “We conducted a prospective … age 18 – 40 years old.” Please revisit this statement as this study did not conduct an actual prospective study

Methodology - The methods section requires more details to ensure clarity

1. Please justify the rationale for using the data from 2 cohorts. If I understand correctly from the methodology section, the FAMILY cohort consisted of multiple ethnic groups, including the South East Asian and South Asian. Please justify why only White Europeans were selected from FAMILY, why this study only included the findings from the FAMILY cohort as it consisted of multiple ethnic groups – and further comparison in the associations between red meat intake and GDM among ethnic groups can be made. Besides, there is a time difference between START (2011-2015) and FAMILY cohorts (2004-2009), whereby the eating patterns were somehow different.
2. Supplement figure 1: Please give more clear description regarding the flow of participants

(Consort diagram), some parts of the box cannot be read.

3. Line 196: what type of intake? Total energy intake? Red meat intake?
4. I am wondering why the authors use logistic regression for the cohort study since relative risk (RR), and hazard ratio (HR) are more suitable for conducting statistical analysis. Furthermore, previous studies (lines 85 – 91 & lines 277 - 285) also used RR to perform the analysis. Suggest setting the “medium” group as the reference group. Please confirm and change the statistical analysis.
5. I am confused about how the tertiles (low, medium and high) are determined. Detailed grouping information needs to be provided (e.g., the cut-off for each group). Were those in the low red meat group indeed had low red meat intakes compared to the previous study or standard reference?
6. Line 208 – 216: Further clarification of the multivariate logistic regression is also required. Please explain how the sequence of covariates of the model was made

Results

1. Tables: Please refer to the journal guideline on the presentation of tables.
2. Table 1: Please include n (%) for each category.
3. Table 2: Diet index (last row). Diet quality index (please use a standardized term throughout the entire manuscript)? If not, please include a description in the Methodology section
4. Suggest combining paragraphs 1 and 2 of the results and simplifying it
5. Line 240 – 247: Please revisit this paragraph, as it is confusing.
6. Line 248-259: Please revisit this paragraph, as it did not reflect all the findings in Table 2

Discussion

1. Line 270-272: This finding was not shown in the result section.
2. Line 295-299: Could the authors comment on why white Europeans had higher intakes of refined grains, and lower intakes of fiber (based on 299 a 2000 kcal diet) than South Asians, but had a lower prevalence of GDM?
3. Line 324: what is the cut-off for moderation spoused by most dietetic and health organizations, and are any references for this?
